# When are declines in condom use while using PrEP a concern? Modelling insights from a Hillbrow, South Africa case study

**DOI:** 10.7448/IAS.20.1.21744

**Published:** 2017-09-20

**Authors:** Hannah Grant, Zindoga Mukandavire, Robyn Eakle, Holly Prudden, Gabriela B Gomez, Helen Rees, Charlotte Watts

**Affiliations:** ^a^ Faculty of Public Health and Policy, London School of Hygiene and Tropical Medicine, London, UK; ^b^ Wits Reproductive Health and HIV Institute, University of the Witwatersrand, Johannesburg, South Africa; ^c^ Amsterdam Institute for Global Health and Development, Department of Global Health, Academic Medical Center, University of Amsterdam, Amsterdam, The Netherlands

**Keywords:** HIV, pre-exposure prophylaxis, sexually transmitted infections, mathematical models, behaviour change, risk compensation, condom migration

## Abstract

**Introduction** **:** Oral pre-exposure prophylaxis (PrEP) is a promising new prevention approach for those most at risk of HIV infection. However, there are concerns that behavioural disinhibition, specifically reductions in condom use, might limit PrEP’s protective effect. This study uses the case of female sex workers (FSWs) in Johannesburg, South Africa, to assess whether decreased levels of condom use following the introduction of PrEP may limit HIV risk reduction.

**Methods:** We developed a static model of HIV risk and compared HIV-risk estimates before and after the introduction of PrEP to determine the maximum tolerated reductions in condom use with regular partners and clients for HIV risk not to change. The model incorporated the effects of increased STI exposure owing to decreased condom use. Noting that condom use with regular partners is generally low, we also estimated the change in condom use tolerated with clients only, to still achieve 50 and 90% risk reduction on PrEP. The model was parameterized using data from Hillbrow, Johannesburg. Sensitivity analyses were performed to ascertain the robustness of our results.

**Results:** Reductions in condom use could be tolerated by FSWs with lower baseline condom use (65%). For scenarios where 75% PrEP effectiveness is attained, 50% HIV-risk reduction on PrEP would be possible even with 100% reduction in condom use from consistent condom use as high as 70% with clients. Increased exposure to STIs through reductions in condom use had limited effect on the reductions in condom use tolerated for HIV risk not to increase on PrEP.

**Conclusions:** PrEP is likely to be of benefit in reducing HIV risk, even if reductions in condom use do occur. Efforts to promote consistent condom use will be critical for FSWs with high initial levels of condom use, but with challenges in adhering to PrEP.

## Introduction

Oral pre-exposure prophylaxis (PrEP) is a promising approach to HIV prevention. It is hoped that PrEP might become an effective addition to combined HIV prevention and help to significantly reduce HIV risk for vulnerable groups. This would be especially critical for those populations with low ability to negotiate condom use due to gender and societal power imbalances, such as young women in heterosexual relationships [1] and sex workers [2]. Proof of concept has been demonstrated [3] in four out of the six randomized controlled trials conducted to date, in which higher levels of HIV-risk reduction were associated with higher levels of adherence. Open-label extension studies [3–6] have confirmed PrEP’s importance as a prevention tool, with up to 100% risk reduction estimated in the Open Label Extension (OLE) of the iPrEx trial [7] for men and transgender women who have sex with men adhering to PrEP for at least four out of seven doses a week.

Nonetheless, the two randomized controlled trials [8,9] stopped early for futility cited lack of adherence by the study populations as the cause. Additional implementation concerns have been raised, including antiretroviral (ARV) resistance development resulting from sub-optimal drug adherence levels [10], contraindications [11], challenges in acceptability [12], barriers to access and programme retention [13], and behaviour change [14–18]. Noting both the positive trial results as well as implementation concerns, in July 2012 the World Health Organisation (WHO) [19] called for countries to undertake demonstration projects to gain insight into acceptability, patterns of use, and sustainability of PrEP.

Data since gathered has informed WHO’s September 2015 PrEP guidance [19] recommending oral PrEP for all people at substantial risk of HIV (incidence >3 per 100 person years). However, concerns remain [20–24] regarding the potential limiting effects of a particular form of behaviour change – reductions in condom use (condom migration) – on PrEP. Reductions in condom use not only increase the chance of HIV exposure, but also the exposure to and transmission of sexually transmitted infections (STIs). Increased exposure to STIs increases both the susceptibility of an HIV negative partner, as well as the infectiousness of an HIV positive partner, and thereby HIV transmission [25]. Whilst no trial to date has reported decreased condom use, the high rate of pregnancies reported in the trials [12], results of behavioural surveys [26] and qualitative research [13] indicate that efforts to tackle condom migration may need to be considered in the design of PrEP programmes.

In response to these concerns and to inform PrEP programme design, this study examines the extent to which condom migration is likely to impact PrEP effectiveness in programmes for female sex workers (FSWs). We focus on the FSW population working in Hillbrow, Johannesburg, some of whom are participating in a PrEP demonstration programme undertaken by the Wits Reproductive Health and HIV Institute (WRHI) [27]. The FSW populations in this setting present extremely high baseline HIV prevalence (estimated to be up to 72% [28,29]), elevated levels of STIs [30] low levels of condom use with often high HIV risk [31] regular partners, and known challenges in condom negotiation with clients, where in such settings FSWs may receive a quarter of the average price for transactional sex if condoms are insisted upon [17].

Our study aims to inform rapidly changing policy in South Africa where in November 2015, South Africa’s Medicines Control Council approved the use of the fixed-dose combination of TDF/FTC as PrEP [32]. Locally adapted guidelines [33] were published in early 2016 and PrEP was recently included in South Africa’s National Sex Worker HIV Plan (2016–2019) [34]. PrEP roll out for sex workers started in June 2016.

## Methods

This work builds on that in [35], where an adaption of an HIV-risk equation was used to assess microbicides as a new HIV prevention method. This study uses the established Bernoulli model of HIV transmission [36–39] where the probability of the HIV virus being transmitted through each sexual contact is treated as an independent risk event. We employed static rather than dynamic mathematical modelling to obtain clear deductions regarding the contribution of the parameters being explored to HIV risk, and for the derivation of rules of thumb that can be broadly understood and applied to HIV prevention efforts focused on FSWs. Whilst previous studies [37,40,41] have used mathematical modelling to predict the impact of condom migration on the effectiveness of ARV-based microbicides, this is the first study to consider its impact on oral PrEP, in particular for FSWs.

The HIV risk equations for a population of HIV-negative FSWs and their partners prior to, and following introduction of, PrEP are outlined in the *Supplementary Methods*. To explore the consequences for FSWs of condom migration on PrEP, condoms are assumed to be used with consistency that may vary with the introduction of PrEP (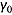 prior to PrEP introduction and 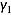 after its introduction). We assumed condoms to have an HIV risk reduction efficacy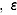, including slippage and breakage. Whilst the risk reduction effectiveness of condoms is generally assumed to follow a linear relationship between use and efficacy (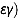, the exact effectiveness relationship between adherence and PrEP efficacy remains under investigation [42–44] (although one study suggested a linear relationship [45]), so we assume an overall level of “PrEP effectiveness”, 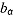, corresponding to a level of FSW PrEP adherence, 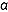. No partner populations are assumed to be taking PrEP.

### Single partner population

We started the analysis by considering a single partner population, in whom the proportion HIV positive is 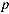. For a given time period, a FSW is assumed to have *n* partners, each with whom she has an average of 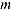 sex acts. For simplicity these equations assume an overall average probability of HIV transmission, 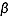, per sexual contact with an HIV-positive partner.

We used the HIV risk equations to derive two key threshold conditions: (1) the level of PrEP effectiveness that must be attained for PrEP to be of benefit in reducing HIV risk, considering any change in condom consistency; and (2) the “break-even” level of condom consistency after introduction of PrEP such that HIV acquisition risk is not increased.

### Single partner population, accounting for increased STI exposure

We expanded our analysis to explore the increased risk of HIV transmission resulting from exposure to STIs, should condom migration occur and PrEP use be inconsistent. *s* is taken as the probability that at least one person in the partnership has an STI, and 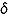 the multiplicative increase in per sex act probability of HIV transmission in the presence of an STI.

We derived the percentage reduction in condom consistency tolerated for HIV risk not to increase on PrEP and compared these results to those not accounting for increased STI exposure, to see whether conclusions remain robust.

### Two partner populations, accounting for increased STI exposure

We then extended the HIV-risk equations to account for risk arising from two distinct partner populations: clients 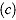 and regular partners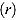. In this setting, condom consistency with regular partners is low [46] and clients sometimes pay more for condom-less sex [47]. As such, any change in condom consistency on PrEP is likely to be more profound with clients, and therefore its impact on HIV risk. We thus examined the percentage reduction in condom consistency *with clients* tolerated for HIV risk not to increase on PrEP, holding condom consistency with regular partners constant (using S*upplementary Materials* equations S14 and S15). We assessed whether the results remain the same, accounting or not for increased STI exposure through decreased condom use. To gauge whether, in such settings, changes in condom use with clients or regular partners present the biggest HIV risk, we assessed whether there is a significant difference in the percentage reduction in condom consistency *with clients* tolerated for HIV risk not to increase, if PrEP use reduces condom use to zero with regular partners.

The equations were solved numerically using Solver in Microsoft Excel 2013 (set to perform 10,000 iterations per calculation) to ascertain the maximum change in condom consistency that can be tolerated for PrEP to remain of benefit, considering increased exposure to STIs, across a range of possible attained PrEP effectiveness levels.

### Data and model parameterization

The HIV-risk equations were parameterized using sexual behaviour data from Hillbrow, Johannesburg collected by WRHI, as well as biological and epidemiological data from other literature (*Supplementary Methods: Table S1*). As there is uncertainty about the PrEP effectiveness corresponding to levels of drug adherence, calculations were carried out for a range of simulated values of PrEP effectiveness for a given adherence value (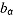). The values simulated roughly span the range of risk reduction estimated through the iPrEx OLE [7] study (between 44% corresponding to fewer than 2 tablets a week and 100% corresponding to at least 4 tablets a week). We started from a slightly lower baseline of 35% to reflect, conservatively, that this study was conducted in a different study population.

It was assumed that all sex acts are peno-vaginal on the basis of available epidemiological data for FSWs in Hillbrow [46]. Three months was chosen as the period of HIV-risk evaluation, as this corresponds to the period after which an HIV test must be performed on PrEP to check for seroconversion (amongst other indicators) [34,48].

### Sensitivity analysis

Two categories of sensitivity analysis were performed. First, the calculations were repeated for two boundary cases: high risk (HR) and low risk (LR) FSWs, parameterized using high- and low-risk values in the HIV-risk equation for the sexual behaviour parameters (% partners HIV positive, number of partners and average number of sex acts per three months, probability at least one person in the partnership has an STI) and the transmission probability parameters (condom HIV-risk reduction efficacy, probability of HIV transmission through peno-vaginal sex, multiplicative increase in per sex act probability of HIV transmission in the presence of an STI).

A second set of sensitivity analyses were undertaken to explore the case that any condom migration brings with it increases in STI prevalence, and therewith risk of HIV transmission. In spite of high levels of STI treatment in the FSW population [49], to obtain conservative results in terms of change in condom consistency tolerated following the introduction of PrEP, we assumed that STIs are present in *all* partnerships where reductions in condom consistency occur, and that these STIs are transmitted through the sex act if not already present in both partners. The probability that at least one person in the partnership has an STI following the introduction of PrEP is therefore assumed to increase at the same rate as the change in condom consistency.

## Results

### Single partner population

We deduced that where the level of PrEP effectiveness achieved equals or exceeds that of condoms (i.e. condom efficacy * baseline condom consistency), PrEP will be of equal or greater benefit in reducing HIV risk and therefore condom use could be reduced to zero without HIV risk increasing. Where the level of PrEP effectiveness is less than the effectiveness originally achieved with condoms, we see that greater drops in condom consistency can be tolerated for those FSW with lower baseline condom consistencies.

**Figure 1.**
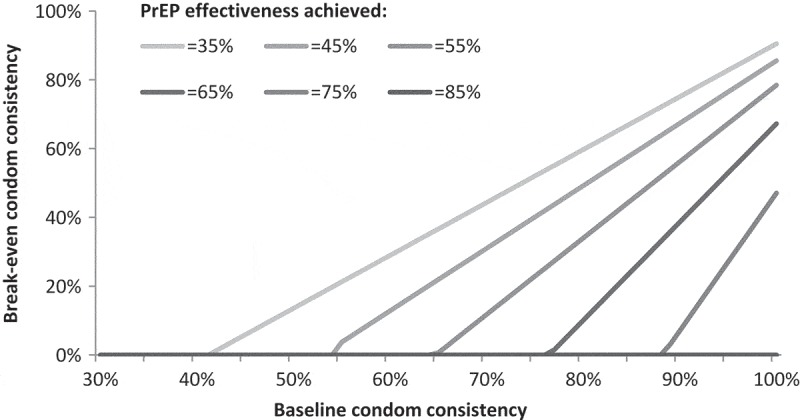
Break-even condom consistencies following introduction of PrEP. In the case of a single partner population, the figure describes the break-even condom consistencies (the levels that condom use could be reduced to, following introduction of PrEP) such that HIV risk is not increased on PrEP. These break-even levels are shown for baseline condom consistencies between 30% and 100%, and corresponding to six different levels of achieved PrEP effectiveness ranging from 35% to 85% (85% corresponding to the level of condom efficacy assumed in this study).

Figure 1 shows the break-even condom consistency after introduction of PrEP such that HIV risk is not increased. Large relative reductions in condom consistency on PrEP are anticipated to be especially well tolerated where higher levels of PrEP effectiveness achieved (>65%). For FSWs whose baseline consistencies are low (<55%), or where there is not anticipated to be a large relative drop in condom consistency on PrEP, even the achievement of low levels of PrEP effectiveness will reduce HIV risk.

### Single partner population, accounting for increased STI exposure

The results show that reductions in condom consistency on PrEP are especially well tolerated for FSWs with lower baseline condom consistencies (<50%) and where higher levels of PrEP effectiveness are achieved (>65%). Even for the lowest level of 35% PrEP effectiveness simulated (which would correspond to adherence to fewer than two tablets a week according to iPrEx OLE [7] estimates), the percentage reduction in condom consistency tolerated steadily increases upwards from a minimum reduction of 17% (corresponding to 90% baseline condom consistency) to 100% migration (corresponding to 30% baseline condom consistency).

Where PrEP effectiveness of 85% can be achieved (which would correspond to adherence of 2–3 tablets a week according to iPrEx OLE [7] estimates; and the exact level of assumed condom protection efficacy simulated for the base case), 100% condom migration can uniformly be tolerated across all baseline condom consistencies simulated.

**Figure 2.**
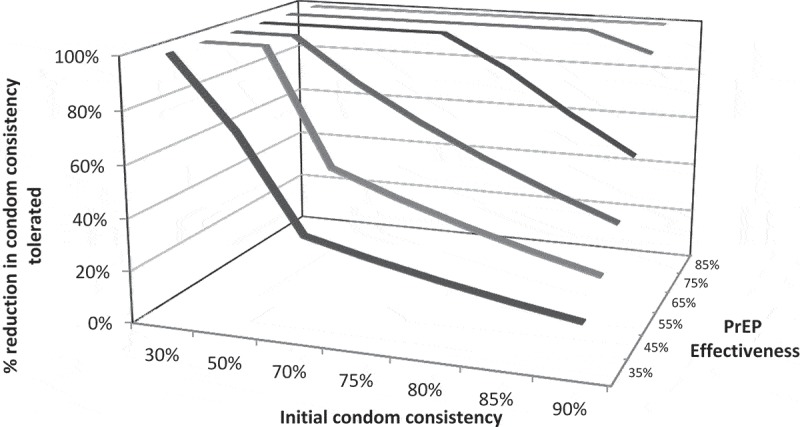
Percentage reduction in condom consistency tolerated with a single partner population for HIV risk not to increase on PrEP, accounting for the effect of STIs on HIV risk. For different baseline condom consistencies between 30 and 95%, the figure describes the percentage reduction in condom consistency that could be tolerated on PrEP corresponding to six different levels of achieved PrEP effectiveness, ranging from 35 to 85% (85% corresponding to the level of condom efficacy assumed in this study).

The percentage change in condom consistency possible is almost the same (<1% difference) whether STIs are accounted for or not in the HIV-risk equations (*Supplementary Results: Table S2*, shown graphically in Figure 2). This is because, whilst inclusion of STI parameters in the mathematical HIV-risk equations does result in increased HIV risk levels on an absolute basis, it does not significantly affect change in risk on a relative basis.

#### Sensitivity analysis

Looking at the boundary cases of high- and low-risk FSW reveals only small variations in the percentage reduction in condom consistency tolerated (accounting for STIs or not in the equations). This is especially true at lower levels of PrEP effectiveness and higher baseline condom consistencies (4–8% reduced reduction in condom consistency tolerated), although this is slightly more pronounced at higher levels of PrEP effectiveness (up to 22% reduced reduction).

Should condom migration brings with it increases in STI prevalence in the population, there would be modest reductions in the percentage reduction in condom consistency tolerated (at most 22% reductions in relative terms compared to the base case results, or between 2 and 20% less in absolute terms), though the differences in the results are smaller especially where PrEP effectiveness achieved is lower (<65%) and initial condom consistency is high (>70%), or where PrEP effectiveness achieved is higher (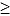
65%) and initial condom consistency is below ~80%.

### Two partner populations, accounting for increased STI exposure

Table 1 demonstrates the percentage reductions in condom consistency with clients tolerated to achieve 50 or 90% levels of reduction in HIV risk on PrEP, condom consistency with regular partners held constant (at 10% [50]).

**Table 1.** Maximum tolerated % reduction in condom consistency with clients (consistency with regular partners held constant) to still achieve 50% or 90% reductions in HIV risk on PrEP, for different levels of PrEP effectiveness achieved

	**55% PrEP Effectiveness**								**75% PrEP Effectiveness**								**95% PrEP Effectiveness**							
	**% reduction in condom consistency with clients tolerated to get overall HIV risk reduction of**																							
	**50%**				**90%**				**50%**				**90%**				**50%**				**90%**			
	Accounting for STIs		Not accounting for STIs		Accounting for STIs		Not accounting for STIs		Accounting for STIs		Not accounting for STIs		Accounting for STIs		Not accounting for STIs		Accounting for STIs		Not accounting for STIs		Accounting for STIs		Not accountingfor STIs	
Initial condom consistency	Base	(LR,HR)	Base	(LR,HR)	Base	(LR,HR)	Base	(LR,HR)	Base	(LR,HR)	Base	(LR,HR)	Base	(LR,HR)	Base	(LR,HR)	Base	(LR,HR)	Base	(LR,HR)	Base	(LR,HR)	Base	(LR,HR)
90%	6%	(−3%,-3%)	6%	(−3%, 3%)	-	-	-	-	57%	(−27%, 26%)	57%	(−28%, 39%)	-	-	-	-	100%	(*,*)	100%	(*,*)	56%	(−27%,19%)	57%	(−28%,37%)
80%	8%	(−3%,-5%)	8%	(−3%,2%)	-	-	-	-	76%	(−31%,24%)	77%	(−31%, 23%)	-	-	-	-	100%	(*,*)	100%	(*,*)	75%	(−29%,20%)	76%	(−31%,24%)
70%	10%	(−3%,-6%)	11%	(−4%,2%)	-	-	-	-	100%	(*,*)	100%	(*,*)	-	-	-	-	100%	(*,*)	100%	(*,*)	100%	(*,*)	100%	(*,*)
50%	18%	(−4%,-13%)	19%	(−5%,2%)	-	-	-	-	100%	(*,*)	100%	(*,*)	-	-	-	-	100%	(*,*)	100%	(*,*)	100%	(*,*)	100%	(*,*)
30%	37%	(−6%,-29%)	38%	(−7%,0%)	-	-	-	-	100%	(*,*)	100%	(*,*)	-	-	-	-	100%	(*,*)	100%	(*,*)	100%	(*,*)	100%	(*,*)

For each level of PrEP effectiveness demonstrated, the table shows the % reduction in condom consistency that could be tolerated, from varying levels of initial condom consistency, to achieve either 50% or 90% HIV-risk reduction. The results are shown for both the case that STIs are accounted for in the HIV risk equations, as well as the case that they are not. The results are shown for the base case parameterization of the model, as well as the boundary cases explored through the first sensitivity analysis of high- and low-risk FSW. They assume that condom consistency with regular partners remains constant at 10% before and after introduction of PrEP. The results corresponding to the case that condom consistency with regular partners drops from 10% to 0% following the introduction of PrEP is shown in Table S4 in the Supplementary Materials. “–” indicates that achievement of the risk reduction is not possible. “*” indicates full migration will still result in higher levels of risk reduction. “Base” refers to the main calculated results undertaken using the baseline parameter values. *HR* stands for high risk and *LR* stands for low risk FSW, and the results calculated in the sensitivity analysis for the boundary parameter cases. A graphic depiction of the results corresponding to achievement of 50% HIV risk reduction on PrEP is given in *Supplementary Equations, Figure S1.*

Achievement of 50% reduction in HIV risk on PrEP is feasible across all simulated PrEP effectiveness levels (55, 75, 95%) and baseline condom consistencies (30–90%). As seen for single partner populations, reductions in condom consistency are best tolerated for FSWs with lower baseline levels with clients or where higher PrEP effectiveness levels are achieved.

A FSW with initial condom consistency of 30% with clients could reduce her consistency by one-third and still achieve 50% reduction in HIV risk, if she were able to attain 55% PrEP effectiveness (corresponding to below 2–3 doses a week per iPrEx OLE [7]). A FSW achieving 95% PrEP effectiveness (corresponding to around 4 doses a week per iPrEx OLE [7]) could tolerate 100% condom migration to achieve HIV risk reductions in excess of 50%; and so too for those FSWs achieving 75% PrEP effectiveness for baseline condom consistencies with clients of up to 70%.

Across the parameters simulated, the higher level of 90% risk reduction on PrEP could only be achieved in the case where PrEP is 95% effective (corresponding to around 4 doses a week per iPrEx OLE [7]). In this case, an initially 90% condom-consistent FSW could reduce her condom use with clients by more than half; and for FSWs with baseline condom consistencies with clients of 70% and lower, 100% condom migration on PrEP could be tolerated.

Again, there is negligible observable (<1%) difference whether or not STIs are accounted for in the HIV risk equations.

In the case that PrEP leads to full condom migration with regular partners, rather than remaining consistent at 10%, there is a small further reduction in condom consistency tolerated (between 1 and 8% across the scenarios simulated, see *Supplementary Results Table S4)*).

#### Sensitivity analysis

Looking at the boundary cases of high- and low-risk FSW reveals small variation in the percentage reduction in condom consistency tolerated for the lower level of PrEP effectiveness of 55%. The variation is more pronounced for higher levels of PrEP effectiveness (75 and 95%), with up to around one-third change in percentage reduction in condom consistency tolerated across the parameter ranges simulated.

In the case that condom migration brings with it increases in STI prevalence in the population, there are reductions in relative terms of 14–26% compared to the base case results, and in absolute terms the reductions are almost uniformly within the range of variation seen through examining the boundary cases of high- and low-risk FSW.

## Discussion

This study provides insights into the risks associated with condom migration following the introduction of PrEP into a comprehensive HIV prevention programme for FSWs. The study demonstrates that the success of PrEP will rest upon its ability to achieve high enough PrEP adherence in FSWs such that the increased protection achieved outweighs the increased HIV risk owing to condom migration and increased STIs exposure. The added value for decision makers of our study lies upon our ability to quantify these trade-offs.

This study has demonstrated that where a FSW’s adherence to PrEP achieves a level of effectiveness that exceeds that of condoms, PrEP will always reduce HIV risk. Condom migration is anticipated to be especially well tolerated where baseline levels of condom consistency are low (<50%) or where a reasonably high level of PrEP effectiveness (>65%) can be achieved. Should FSWs’ condom consistency with regular partners remain low (~10%) or be reduced to zero on PrEP, reductions in condom consistency with clients could uniformly be tolerated whilst still achieving 50% HIV-risk reduction (assuming achieved PrEP effectiveness of at least 55%). This is especially noteworthy having considered probabilities of up to 60% likelihood of STI exposure in a partnership if condom migration were to occur.

From a programming point of view, strategies to identify FSWs with initially higher condom-consistent behaviour but anticipated to adhere less well to PrEP will be important, and efforts to promote condom consistency and give adherence support critical. Considering that full condom migration with regular partners does not substantially increase HIV risk on PrEP (assuming initially low consistency with regular partners holds true), efforts to encourage condom consistency with clients will be critical.

The study has demonstrated that the break-even point at which PrEP is beneficial in terms of HIV-risk reduction is driven primarily by the behavioural parameters of condom consistency and drug adherence, as well as by the efficacy of condoms, and much less by epidemiological parameters. This is noteworthy in programme design, as efforts to improve and sustain behaviours relating to PrEP adherence and condom consistency will have the greatest influence on programme outcomes over epidemiologic context.

There are, however, a number of caveats to the study. This work does not speak to acceptable PrEP adherence levels, given the risk of ARV resistance, noting that PrEP users in the middle adherence spectrum are anticipated to be at greatest risk [51]. This study does not account for a partner’s stage of HIV infection or ARV use in partner populations. The former may increase HIV risk if partners are likely to be recently infected and thus highly viremic, whereas the latter would likely decrease overall risk; however, neither would be expected to impact comparative estimates of change in HIV risk.

Use of a static rather than dynamic model limits the study to an analysis of FSW HIV risk in isolation of the dynamics of infections between FSWs, their partners and clients and in turn to FSWs. These results, whilst suitable to indicate rules of thumb to guide HIV prevention efforts, cannot provide insight into the downstream impact of the intervention and condom migration on the HIV epidemic in South Africa. Finally, the data used to characterize the FSW population in Hillbrow are limited by being self-reported (susceptible to underreporting) and age, as little has been published since the end of the 1990s, when the HIV epidemic was less evolved [52], although studies are underway.

Most importantly, this study indicates that, assuming oral PrEP is proven effective in FSW populations through ongoing trials, in many situations oral PrEP is likely to be of benefit in reducing HIV risk even if behaviour change were to be a programme reality. It provides guidance around the characteristics of FSWs for whom condom migration may be more of an issue (those with initially high levels of condom consistency with clients, anticipated to adhere poorly to PrEP and significantly migrate away from condoms); and those FSWs for whom PrEP is likely to be an important addition to combined HIV prevention measures (those with initially low condom consistency with clients, or anticipated to adhere reasonably well to PrEP). Importantly for the latter group, PrEP will provide additional protection against HIV transmission from regular partners, with whom there is otherwise little protection given low baseline condom levels. Finally, the analytic approach followed in this study could easily be adapted to other vulnerable populations beyond FSW.

## Notes

To access the supplementary material to this article please see Supplementary Files under Article Tools online.
